# To explore the relationship between endometrial hyperemia and polycystic ovary syndrome

**DOI:** 10.1515/biol-2025-1154

**Published:** 2025-09-01

**Authors:** Shuang Wang, Feng-Hua Li, Wei Zhang, Hong-Chu Bao, Cui-Fang Hao

**Affiliations:** School of Medicine, Shandong University, Jinan, Shandong, 250000, China; Reproductive Medicine Centre, Yantai Yuhuangding Hospital, Yantai, Shandong, 264000, China; Reproductive Medicine Centre, Qingdao Women and Children’s Hospital, Qingdao, Shandong, 266000, China

**Keywords:** PCOS, CE, hysteroscopy, hyperemia, HIF-1α, VEGF, ERS

## Abstract

The aim of this study is to investigate the characteristics and etiology of endometrial hyperemia in patients with polycystic ovary syndrome (PCOS) through two complementary approaches: clinical data analysis to characterize endometrial hyperemia and clinical trials to elucidate its underlying causes. ELISA was employed to quantify inflammatory mediators in endometrial tissue, while reverse transcription quantitative polymerase chain reaction (RT-qPCR) and Western blot analyses were conducted to assess the expression levels of molecules associated with endoplasmic reticulum stress (ERS). Additionally, RT-qPCR was used to determine the mRNA expression levels of HIF-1α, VEGF, and EPO. Compared with non-PCOS patients, those with PCOS exhibited a significantly higher prevalence of chronic endometritis (CE) (*P* < 0.05) along with increased levels of inflammatory factors (*P* < 0.05). Furthermore, the mRNA expression levels of HIF-1α, VEGF, and EPO, as well as ERS-related molecules, were significantly elevated in PCOS patients (*P* < 0.05). These findings indicate that women with PCOS are more likely to suffer from CE and that endometrial hyperemia is the primary manifestation of CE in these patients. The results further suggest that endometrial hypoxia-induced ERS may contribute to the development of endometrial hyperemia in PCOS patients.

## 1 Introduction

Polycystic ovary syndrome (PCOS) is the most prevalent gynecological endocrine and metabolic disorder affecting women, particularly during the reproductive years, with an incidence of approximately 5–8% [1,2]. Clinically, PCOS is characterized by menstrual irregularities, polycystic ovarian morphology, infertility, and metabolic disturbances such as obesity and insulin resistance, which constitute significant risk factors for cardiovascular diseases and endometrial cancer. Chronic endometritis (CE) is a persistent inflammation of the endometrium with diverse, nonspecific manifestations. Often asymptomatic or presenting with mild symptoms – such as abnormal uterine bleeding, atypical vaginal discharge, and dull abdominal pain – CE is frequently underdiagnosed in clinical practice. CE is commonly identified during hysteroscopic examinations or through histopathological analysis. Hysteroscopic findings of CE may include focal or diffuse endometrial hyperemia, the presence of endometrial micropolyps (less than 1 mm), and stromal edema with hyperplasia. The pathological hallmark of CE is the infiltration of plasma cells within the endometrial stroma, a diagnosis that may also be confirmed by positive immunohistochemical staining for CD138 [3,4]. CE adversely affects endometrial receptivity, contributing to reduced pregnancy rates, repeated implantation failure, recurrent miscarriage, and related reproductive complications [5,6].

Throughout our long-term clinical work, we observed that a substantial proportion of patients with PCOS exhibited diffuse endometrial hyperemia during hysteroscopy, a manifestation consistent with CE. A comprehensive review of the literature, however, did not reveal any studies directly examining the relationship between PCOS and CE. Moreover, recent investigations have demonstrated that women with PCOS often display impaired endometrial receptivity, adversely affecting pregnancy outcomes [7,8]. This raises the question of whether endometrial hyperemia might negatively influence endometrial receptivity in patients with PCOS. Given the complex pathological and physiological characteristics of PCOS, its specific pathogenesis remains incompletely understood, with most studies primarily focusing on ovulatory dysfunction and metabolic abnormalities while paying relatively little attention to the endometrium. Consequently, our research was designed to address two key aspects: first, by performing a statistical analysis of clinical data to elucidate the correlation between PCOS and CE; and second, by investigating the potential molecular mechanisms underlying endometrial hyperemia in PCOS patients.

Obesity is a major risk factor for PCOS and represents its most common comorbidity [9]. Adipocyte hypertrophy contributes to the excessive expansion of adipose tissue; however, when neovascularization does not keep pace with this growth, localized hypoxia ensues. In a hypoxic environment, cellular defense mechanisms are activated to promote cell survival. Research indicates that these defense mechanisms are mediated by specific transcription factors, such as HIF-1α and VEGF, which orchestrate the cellular response to low oxygen conditions. HIF-1α plays a critical role in regulating angiogenesis, erythropoiesis, inflammation, and glucose metabolism [10]. Under hypoxic conditions, adipose tissue may experience macrophage infiltration, resulting in the substantial secretion of inflammatory mediators including IL-1β, IL-6, IL-18, and TNF-α. It is conceivable that hypoxia contributes to endometrial hyperemia in patients with PCOS. Moreover, various stressors – such as hypoxia, nutrient deprivation, and calcium ion imbalance – can disrupt endoplasmic reticulum (ER) function, leading to the accumulation of unfolded or misfolded proteins, a condition known as ER stress (ERS). Although ERS initially constitutes a protective response in eukaryotic cells, prolonged ERS can precipitate apoptosis. Recent studies have demonstrated a close interrelationship between inflammation and ERS; specifically, ERS can upregulate pro-inflammatory factors, including IL-6 and TNF-α, thereby perpetuating a cycle of inflammation [11]. These findings suggest that ERS and inflammatory pathways interact through multiple mechanisms, contributing to the onset and progression of various inflammatory diseases [12].

PCOS is a complex disease. It is hoped that this study can provide some new ideas for the treatment of patients with PCOS.

## 2 Materials and methods

### 2.1 Study subjects

A retrospective review was conducted on infertility patients treated with assisted reproduction in the Reproductive Medicine Department at Yuhuangding Hospital in Yantai. Between 2017 and 2019, a total of 2,498 female patients, with an average age of 31.45 ± 3.55 years, underwent hysteroscopic evaluation; among these, 261 patients were diagnosed with PCOS. The inclusion criteria were as follows: (1) fulfillment of the diagnostic criteria for infertility; (2) age ≤ 40 years; (3) infertility attributed to ovulatory disorders, tubal factors, or male factors; and (4) for patients with PCOS, compliance with the “Chinese Guidelines for the Diagnosis and Treatment of Polycystic Ovary Syndrome” published in 2018 by the Gynecological Endocrinology Group of the Obstetrics and Gynecology Branch of the Chinese Medical Association [13]. The exclusion criteria included: (1) concurrent conditions such as endometriosis or hydrosalpinx; (2) acute inflammatory diseases of the reproductive tract, such as vaginitis or pelvic inflammatory disease; and (3) premature ovarian failure. Clinical data were collected based on these specified inclusion and exclusion criteria.


**Informed consent:** Informed consent has been obtained from all individuals included in this study.
**Ethical approval:** The research related to human use has been complied with all the relevant national regulations, institutional policies and in accordance with the tenets of the Helsinki Declaration, and has been approved by the Ethics Committee of Yuhuangding Hospital, Yantai (Approval number: [2020] No. (06)).

## 3 Research methods

**Figure 1:**
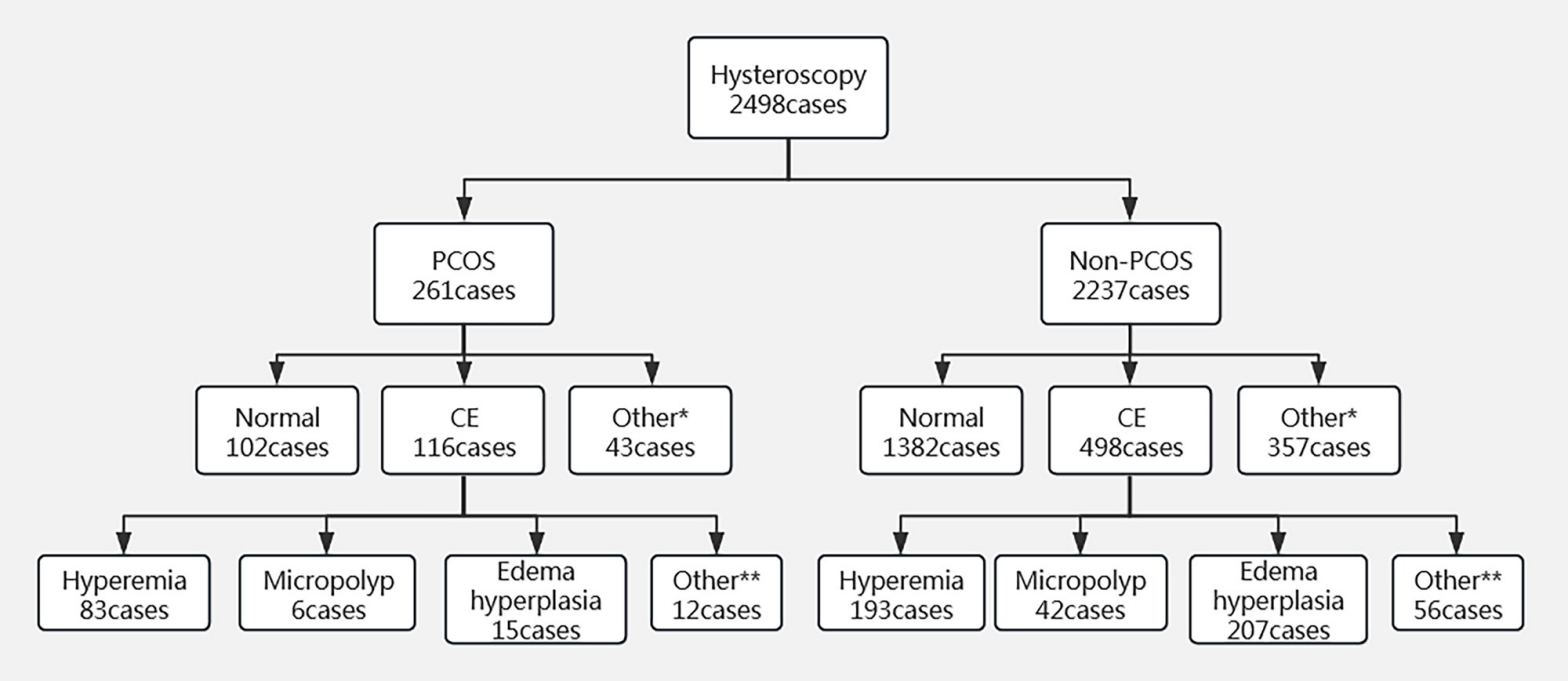
Grouping flowchart. Notes: Other* refers to endometrial polyps, uterine submucous myoma, uterine adhesions, abnormal endometrial hyperplasia, unicornous uterus, uterine mediastinum and other abnormal uterine cavity; Other** refers to two or more manifestations of endometrial inflammation at the same time.

Based on hysteroscopic findings, both PCOS and non‐PCOS patients were classified into three groups: a normal group, a CE group, and a miscellaneous group* comprising patients with endometrial polyps, uterine submucosal myoma, uterine adhesions, atypical endometrial hyperplasia, unicornuate uterus, mediastinal uterus, and other abnormal uterine cavity conditions. The CE group was further subdivided into the hyperemia group, the micropolyp group, the edema‐hyperplasia group, and an additional group** consisting of cases with two or more concurrent manifestations of endometrial inflammation. The study grouping is illustrated in Figure 1. *Other groups include endometrial polyps, uterine submucous myoma, uterine adhesions, abnormal endometrial hyperplasia, unicornuate uterus, mediastinal uterus, and other abnormal uterine cavity conditions. **Other group refers to cases with two or more simultaneous manifestations of endometrial inflammation.

### 3.1 Hysteroscopy examination process

**Figure 2:**
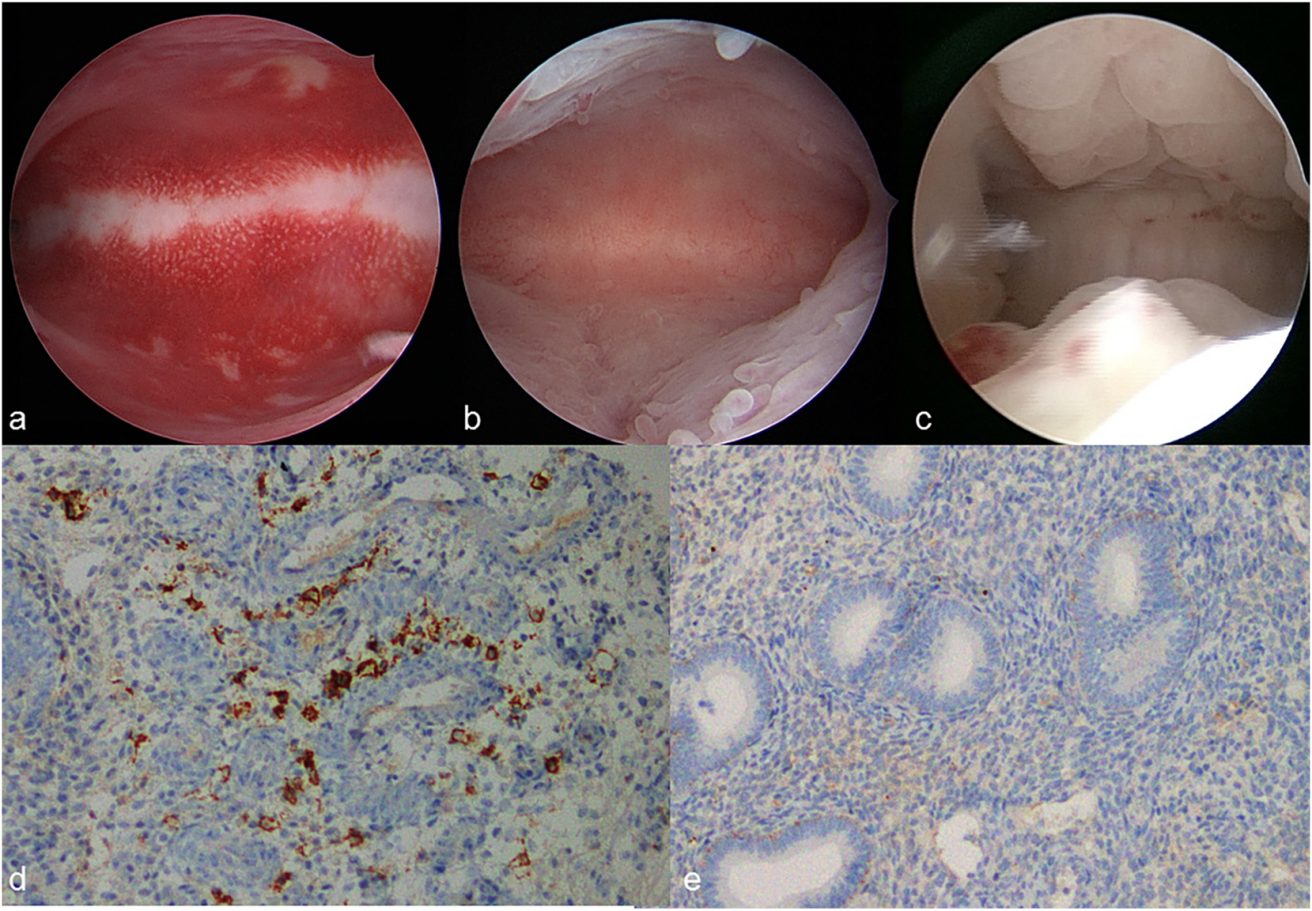
Different features of chronic endometritis under fluid hysteroscope. Note: (a) Hyperemic endometrium. (b) Micropolyps (less than 1 mm in size). (c) Edema and hyperplasia. (d) Positive CD138 IHC staining (brown color), and >5 plasma cells in the endometrial stroma were positively stained with CD138 antibody (×400 pi). (e) Negative CD138 IHC staining, and <5 plasma cells were found in the endometrial stroma (×400 pi). pi = pixels per inch.

At 3–5 days after menstruation, all patients underwent gynecological and vaginal discharge examinations to rule out contraindications for hysteroscopy, such as vaginitis and pelvic inflammatory disease. Hysteroscopy was performed using a lens-based mini-telescope (Karl Storz, Tuttlingen, Germany; outer diameter: 2.7 mm; viewing angle: 105°; operative sheath with double-flow channels: 4.5 mm). A 0.9% saline solution was used to distend the uterine cavity at an inflation pressure of 80–100 mmHg. The procedure employed a 300 W light source paired with a high-definition digital camera and a xenon bulb (Karl Storz, Tuttlingen, Germany). Outcome measures included the shape of the uterine cavity, and the color, thickness, elasticity, smoothness, and glandular appearance of the endometrial surface, as well as the configuration of the fallopian tube openings. Clinical endometritis was diagnosed based on the presence of generalized periglandular hyperemia resembling “strawberry spots,” isolated or diffuse micropolyps, and stromal edema with hyperplasia (Figure 2). All hysteroscopic examinations were performed by the same physician to minimize variations.

### 3.2 Endometrial specimen collection

This study received approval from the Ethics Committee of Yuhuangding Hospital in Yantai (Approval Number: [2020] No. (06)), and informed consent was obtained from all enrolled patients. The investigation adhered to the guidelines set forth in the STROBE Statement. The CE group was characterized by microplasia and positive CD138 immunohistochemistry (IHC), whereas the PCOS group exhibited endometrial hyperemia with negative CD138 IHC results, and the control group presented with a normal endometrium and negative CD138 IHC. Each cohort consisted of ten cases. Table 1 delineates the clinical characteristics of the three groups.

**Table 1:** Clinical characteristics of the three groups of patients

General features	Control group (*N* = 10)	CE group (*N* = 10)	PCOS group (*N* = 10)	*F*	*P* value
Age (year)	33.40 ± 2.72	32.00 ± 3.74	32.00 ± 2.31	0.73	0.49
Duration of infertility (year)	4.35 ± 2.33	3.85 ± 2.38	4.30 ± 3.65	0.09	0.91
BMI (kg/m^2^)	19.97 ± 1.96	21.88 ± 1.87	26.97 ± 3.10*	23.18	<0.001
bFSH (UI/L)	6.08 ± 1.84	6.19 ± 1.10	5.75 ± 1.24	0.25	0.78
bLH (UI/L)	4.55 ± 2.70	4.46 ± 1.27	8.69 ± 5.28*	4.75	0.02
bE_2_ (pg/mL)	32.01 ± 11.99	28.88 ± 12.08	31.43 ± 12.63	0.19	0.83
bT (ng/mL)	0.23 ± 0.09	0.22 ± 0.09	0.37 ± 0.15*	4.91	0.02
bPRL (ng/mL)	16.00 ± 6.52	18.73 ± 6.89	17.38 ± 7.75	0.37	0.69
AMH (ng/mL)	3.03 ± 1.19	2.21 ± 0.92	11.30 ± 6.10*	19.19	<0.001
CA125 (U/mL)	25.08 ± 8.94	20.35 ± 9.38	17.88 ± 8.30	1.70	0.02

Notes: *P* value < 0.05 indicates a significant difference. * indicates that there is a statistically significant difference between the PCOS group and the CE group as well as the control group, and the difference is consistent with the clinical characteristics of PCOS patients.

### 3.3 Endometrial tissue oil red O staining

Frozen sections of endometrial tissue were initially rinsed with distilled water and then soaked in 60% isopropyl alcohol for 2 min. The sections were subsequently stained with an Oil Red O working solution for 2–5 min and counterstained with 60% isopropyl alcohol for an additional 2 min. After a water rinse, the tissue was re-stained with hematoxylin for 1 min, differentiated in hydrochloric acid ethanol for 2 s, and blued with running water before being sealed with glycerin gelatin. The area ratio of lipid droplets was quantitatively analyzed using Image-Pro Plus 6.0 software.

### 3.4 ELISA was used to detect the concentration of inflammatory factors (IL-1β, IL-6, IL-18, and TNF-α) in endometrial tissue

A double-antibody one-step sandwich ELISA kit (Abcam, Cambridge, UK) was employed. Samples, standard products, and HRP-labeled detection antibodies were added to micro-wells pre-coated with interleukin-capturing antibodies, followed by incubation and thorough washing. The substrate TMB generated a blue chromogenic reaction catalyzed by peroxidase, which subsequently turned yellow upon the addition of acid. The resulting color intensity was positively correlated with the interleukin concentration in the sample. Absorbance was measured at a wavelength of 450 nm using an enzyme-labeled instrument, from which the sample concentration was calculated.

### 3.5 RT-qPCR was used to detect the expression level of ATF4, GRP78, HSP70, HIF-1α, VEGF, and EPO mRNA in endometrial tissue

RNA was extracted using the RNeasy Micro Kit (Takara, Osaka, Japan). RNA concentrations were determined by ultraviolet spectrophotometry and subsequently reverse-transcribed into cDNA. The PCR primers are listed in Table 2. The mRNA expression levels of ERS-related molecules ATF4, GRP78, and HSP70 were quantified using specific primers (Qiagen, Germany). To enhance experimental precision, minimize errors during liquid handling, and improve amplification consistency, GAPDH was selected as the internal reference. Amplification reactions were performed on a StepOnePlus™ Real-Time PCR System (Thermo Fisher Scientific), and gene expression levels were calculated using the ∆∆Ct method. The PCR efficiency for all amplicons ranged from 90 to 100%, and all experiments were conducted in triplicate.

**Table 2:** PCR primers

Gene	Primer sequence forward (5′–3′)
ATF4	F-ATGACCGAAATGAGCTTCCTG
	R-GCTGGAGAACCCATGAGGT
GRP78	F-CATCACGCCGTCCTATGTCG
	R-CGTCAAAGACCGTGTTCTCG
HSP70	F-GCATCGAGACTATCGCTAATGAG
	R-TGCAAGGTTAGATTTTTCTGCCT
HIF-1α	F-GAACGTCGAAAAGAAAAGTCTCG
	R-CCTTATCAAGATGCGAACTCACA
VEGF	F-AGGGCAGAATCATCACGAAGT
	R-AGGGTCTCGATTGGATGGCA
EPO	F-GGAGGCCGAGAATATCACGAC
	R-CCCTGCCAGACTTCTACGG
GAPDH	F-GGGAAACTGTGGCGTGAT
	R-GAGTGGGTGTCGCTGTTGA

qPCR reaction was performed at Rotor-GeneTM 6000 (Corbett Research). The mRNA expression level was quantitatively analyzed by 2^−ΔΔCt^.

The protein expression levels of ERS-related molecules, including protein kinase R-like endoplasmic reticulum kinase (PERK), activating transcription factor 4 (ATF4), and CCAAT-enhancer-binding protein homologous protein (CHOP), were determined via Western blot analysis.

Endometrial tissue was homogenized with RIPA lysis buffer in an ice bath, and the supernatant was collected following centrifugation. Total protein concentration was determined using the BCA assay, and the proteins were subsequently separated by SDS-PAGE employing a 4% stacking gel. After electrophoresis, the proteins were transferred onto a cellulose nitrate membrane. The membrane was blocked with skim milk powder at room temperature for 1 h, rinsed with TBST, and incubated overnight at 4°C with primary antibodies targeting PERK, ATF4, and CHOP. Following an additional TBST wash, the membrane was incubated with HRP-conjugated secondary antibodies for 1 h at room temperature. Finally, after a further TBST rinse, the membrane was treated with an electro chemical luminescence hypersensitive luminescent solution in a dark room, and the gray values of the bands were quantified using ImageJ.

### 3.6 Statistical analysis

All analyses were performed using SPSS version 26.0. Categorical variables were reported as proportions (percentages) and compared between groups using either the chi-squared test or Fisher’s exact test. Continuous variables with a normal distribution were expressed as mean value ± standard deviation and compared using the *t*-test, while those that did not conform to normality were presented as medians with the 25th and 75th percentiles [*M*(P25, P75)] and analyzed with the Mann–Whitney *U* test. To enhance comparability between groups and adjust for sample size differences, propensity score matching (PSM) was implemented using a 1:1 no-replacement matching method with a caliper of 0.02. The matching variables included female age, duration of infertility, and basal follicle stimulating hormone (bFSH) levels. A *p*-value of less than 0.05 was considered statistically significant.

## 4 Results

### 4.1 Clinical parameters in the PCOS Group and the non-PCOS Group

A retrospective analysis was performed on patients who underwent assisted reproductive therapy between 2017 and 2019. Based on predefined inclusion and exclusion criteria, a total of 2,498 patients were evaluated. Among these, 261 patients were diagnosed with PCOS, of whom 116 (44.44%) exhibited CE on hysteroscopic examination. In contrast, 498 (22.26%) of the 2,237 non‑PCOS patients were found to have CE by hysteroscopy. Furthermore, the non‑PCOS group showed significantly higher values in terms of age, duration of infertility, and bFSH compared to the PCOS group (*P* = 0.031, 0.036, and 0.004, respectively).

PSM was employed to address the substantial sample size disparity between the PCOS and non-PCOS groups and to control for potential confounding factors. The nearest neighbor matching method was applied using age, duration of infertility, and bFSH as matching variables. A total of 69 pairs were precisely matched, 169 pairs were approximately matched, and 238 pairs were successfully matched. Post-PSM analysis indicated no significant differences in age, duration of infertility, or bFSH between the two groups (all *P* > 0.05). In comparison to non-PCOS patients, those with PCOS presented with increased body mass index (BMI), serum basal luteinizing hormone, serum basal testosterone, and anti-Mullerian hormone levels, as well as a higher number of oocytes retrieved and a greater prevalence of CE. Additionally, PCOS patients exhibited a significantly lower clinical pregnancy rate and a higher abortion rate (*P* < 0.05).

### 4.2 Comparison of hysteroscopy results in PCOS patients and non-PCOS patients

**Figure 3:**
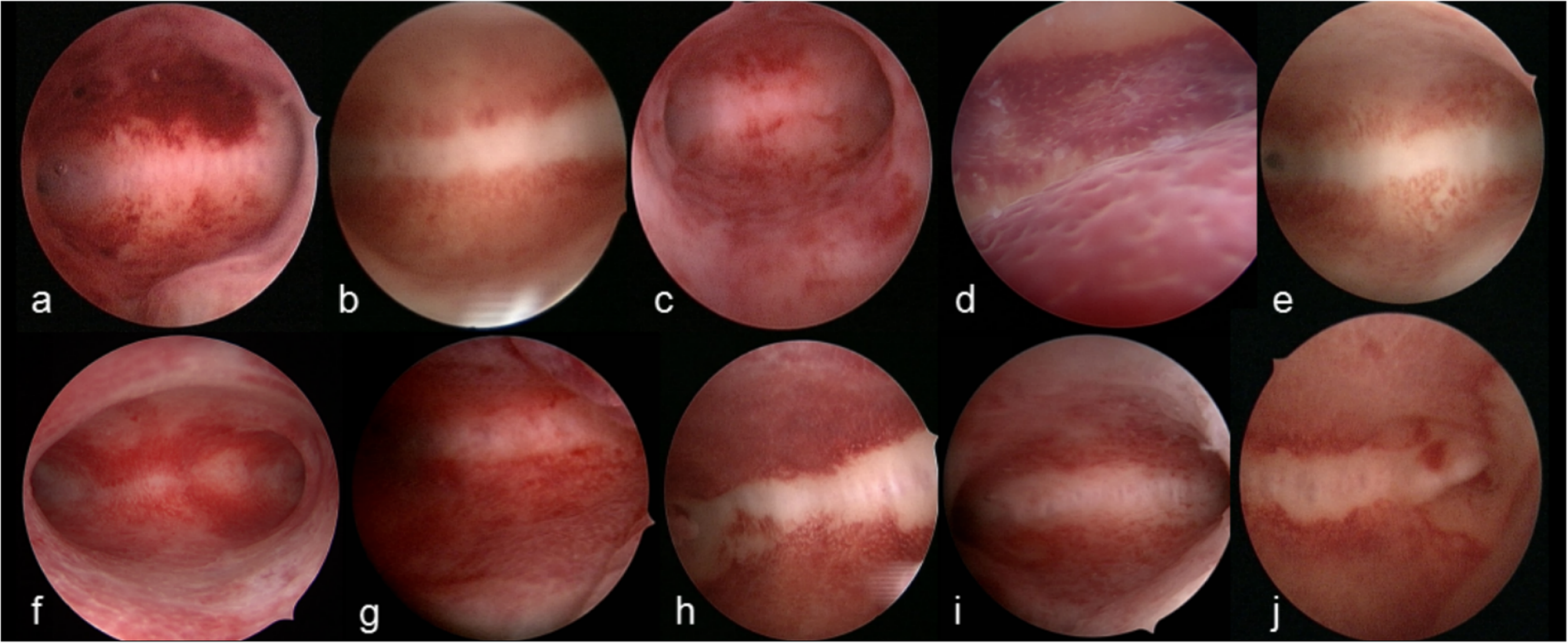
Images (a) to (j) show endometrial hyperemia. Notes: Diffuse endometrial hyperemia with large areas of hyperemic endometrium, sometimes flflushed with what resembles “strawberry spots”; the area is more than half of the uterine cavity.

In this study, CE was observed in 44.44% (116/261) of women with PCOS compared to 22.26% (498/2,237) of non-PCOS women, a difference that was statistically significant (*P* < 0.05). The hysteroscopic manifestations of CE varied between the PCOS and non-PCOS groups. Notably, endometrial hyperemia was significantly more prevalent in PCOS patients (*P* < 0.05), whereas there was no significant difference in the incidence of micropolyps between the groups. In contrast, endometrial edema hyperplasia was more common in non-PCOS patients (*P* < 0.05). Detailed findings are presented in Table 3. Additionally, during hysteroscopic examinations, the endometrium in PCOS patients occasionally exhibited a “flaky hyperemia” pattern, commonly referred to as the “strawberry sign” (Figure 3).

**Table 3:** Clinical characteristics of PCOS patients and non-PCOS patients after PSM [ \[(\bar{x}\pm \text{}s)]\] , *M*(P25, P75)]

General features	PCOS (*n* = 238)	Non-PCOS (*n* = 238)	*P*-value
Age (year)	32.32 ± 3.05	32.09 ± 3.11	NS
Duration of infertility (year)	4.04 ± 2.43	3.98 ± 2.54	NS
BMI (kg/m^2^)	25.59 ± 1.57	22.81 ± 1.83	0.03
bFSH (UI/L)	6.73 ± 1.64	6.94 ± 1.82	NS
bLH (UI/L)	8.29 (5.05, 12.53)	4.76 (3.66, 6.19)	<0.001
bT (ng/mL)	0.37 ± 0.16	0.25 ± 0.17	0.01
AMH (ng/mL)	9.07 (5.85, 13.65)	3.48 (2.08, 5.48)	<0.001
Prevalence of CE (%)	107/238 (44.96)	49/238 (20.59)	<0.001
Oocytes (*n*)	11.66 ± 6.09	9.53 ± 5.08	0.01
Percentage of good quality embryo (%)	1,171/1,837 (63.75)	958/1,493 (64.16)	NS
No. of embryos transferred	1.88 ± 0.33	1.90 ± 0.30	NS
Endometrium thickness on ET day (cm)	1.05 ± 0.17	1.07 ± 0.25	NS
Clinical pregnancy rate (%)	46/112 (41.07)	72/123 (58.53)	0.01
Early pregnancy loss rate (%)	10/46 (21.74)	6/72 (8.33)	0.04

Notes: *P* value < 0.05 indicates a significant difference. NS indicates no statistical significance.

The prevalence of CE was significantly higher among women with PCOS (44.44%) compared to the non-PCOS population (22.26%). In the PCOS cohort, CE predominantly manifested as endometrial hyperemia, a finding that reached statistical significance. Notably, the positive rate of CD138 IHC in patients exhibiting endometrial hyperemia was only 10.14%, which is significantly lower than the rates observed in the micropolyp (62.50%) and edema combined with hyperplasia groups (73.87%). Detailed results are presented in Table 4.

**Table 4:** Hysteroscopic results in PCOS and non-PCOS patients

CE under hysteroscopy	PCOS and CE (*N* = 116)	Non-PCOS and CE (*N* = 498)	*P*-value
Hyperemia	83 (71.55%)	193 (38.76%)	<0.001
Micropolyp	6 (5.17%)	42 (8.43%)	0.239
Edema and Hyperplasia	15 (12.93%)	207 (41.57%)	<0.001
Others*	12 (10.34%)	56 (11.24%)	0.781

Notes: *P* value < 0.05 indicates a significant difference. Others* refers to the simultaneous occurrence of two or more manifestations of endometrial inflammation.

A chi-square test was conducted to compare the positive rates of CD138 IHC among the three groups. The analysis revealed a statistically significant difference in the positive rates (*χ* ^2^ = 166.245, *P* < 0.001). Subsequent pairwise comparisons indicated that the hyperemia group exhibited a significantly lower positive rate than both the micropolyp group and the edema hyperplasia group; however, no significant difference was observed between the micropolyp and edema hyperplasia groups. For further details, please refer to Table 5.

**Table 5:** CD138 IHC positive rate of CE under hysteroscopy

Groups	Hyperemia (*N* = 276)	Micropolyp (*N* = 48)	Edema and hyperplasia (*N* = 222)	*P*-value
CD138 IHC positive rate (%)	28 (10.14%)	30 (62.50%)	164 (73.87%)	<0.001

Note: *P* value < 0.05 indicates a significant difference.

Next clinical trials were conducted to elucidate the cause of endometrial hyperemia in patients with PCOS. Multiple literature reviews revealed that PCOS represents a significant risk factor for the development of preeclampsia [14,15]. The pathogenic process appears to involve insufficient remodeling of the uterine spiral arterioles, excessive activation of inflammatory responses, and damage to vascular endothelial cells. Furthermore, placental ischemia and hypoxia are suggested to play critical roles in this context [16]. Elevated levels of HIF-1α have also been observed in patients with preeclampsia [17]. Motivated by these findings, we initiated experimental approaches focusing on endometrial hypoxia as a novel avenue for investigation.

Next we examined obesity and hypoxia as key factors to advance further research in this field. Clinical data indicated that women with PCOS had significant BMIs than those without PCOS. Initially, we conducted a preliminary experiment to assess the fat content in the endometrial tissue.

### 4.3 Lipid distribution in endometrial tissue sections

**Figure 4:**
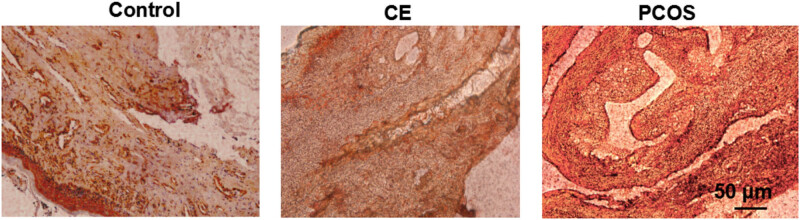
Lipid distribution in endometrial tissue sections. Notes: Compared with the CE group and the control group, the proportion of neutral lipid droplets in endometrial epithelium and interstitial tissue of PCOS group was significantly increased. *P* < 0.05.

Lipid distribution in endometrial tissue sections was evaluated using oil red O staining. The area ratio of lipid droplets was quantified with Image-Pro Plus 6.0, and each experimental group comprised ten cases. The staining results demonstrated that, compared with both the CE and control groups, the PCOS group exhibited a statistically significant increase (*P* < 0.05) in the proportion of neutral lipid droplets within the endometrial epithelium and interstitial tissue, as well as an increase in both the volume and number of lipid droplets. These findings suggest that the overall lipid content in the endometrial tissue of the PCOS group was markedly higher than that observed in the CE and control groups. No significant differences in endometrial lipid distribution were noted between the CE and control groups (Figure 4).

### 4.4 mRNA expression levels of HIF-1α, VEGF, and EPO in endometrial tissues

**Figure 5:**
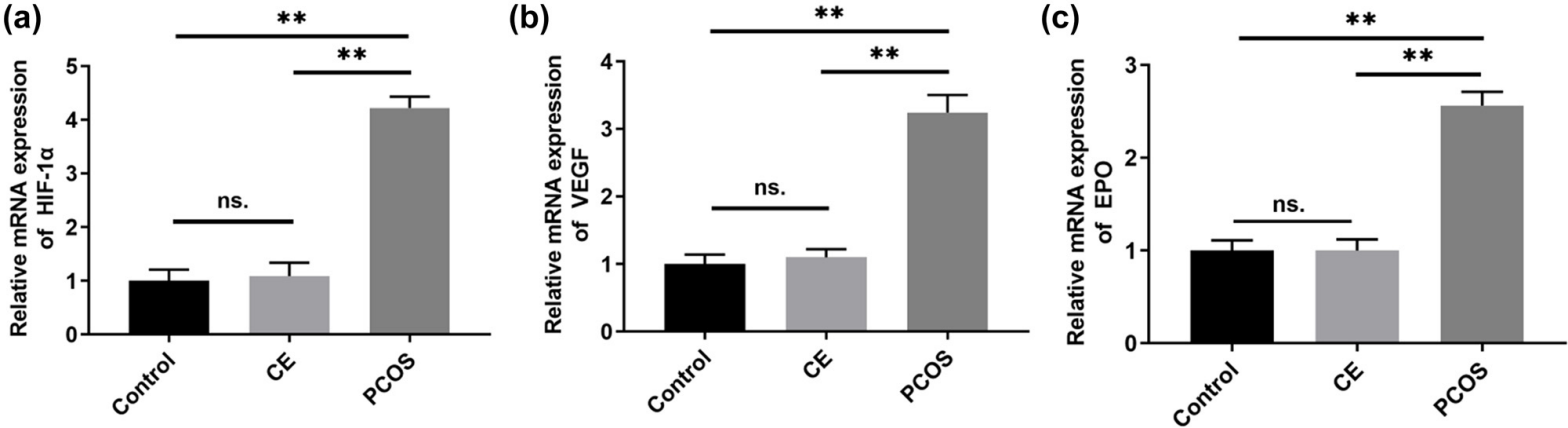
mRNA expression levels of HIF-1α, VEGF and EPO in endometrial tissues. The control group is black (*n* = 10), the CE group is light grey (*n* = 10) and the PCOS group is dark gray (*n* = 10). **P < 0.01. (a) Mean values (bars) ± SD (whiskers) indices mRNA expression levels of HIF-1α in endometrial tissues. (b) Mean values (bars) ± SD (whiskers) indices mRNA expression levels of VEGF in endometrial tissues. (c) Mean values (bars) ± SD (whiskers) indices mRNA expression levels of EPO in endometrial tissues.

RT-qPCR results revealed that, relative to the control group, the mRNA expression levels of HIF-1α, VEGF, and EPO in the endometrial tissues of patients with CE did not exhibit statistically significant differences (*P* > 0.05). In contrast, these expression levels were significantly elevated in the endometrial tissues of patients with PCOS compared to controls (*P* < 0.01). Additionally, when comparing the CE and PCOS groups, PCOS patients showed a marked increase in the mRNA expression levels of HIF-1α, VEGF, and EPO (*P* < 0.01). Each group consisted of ten cases (Figure 5). Subsequently, the concentration of inflammatory factors in endometrial cells was measured to assess the presence of chronic inflammation.

### 4.5 Levels of inflammatory cytokines in endometrial tissue

**Figure 6:**
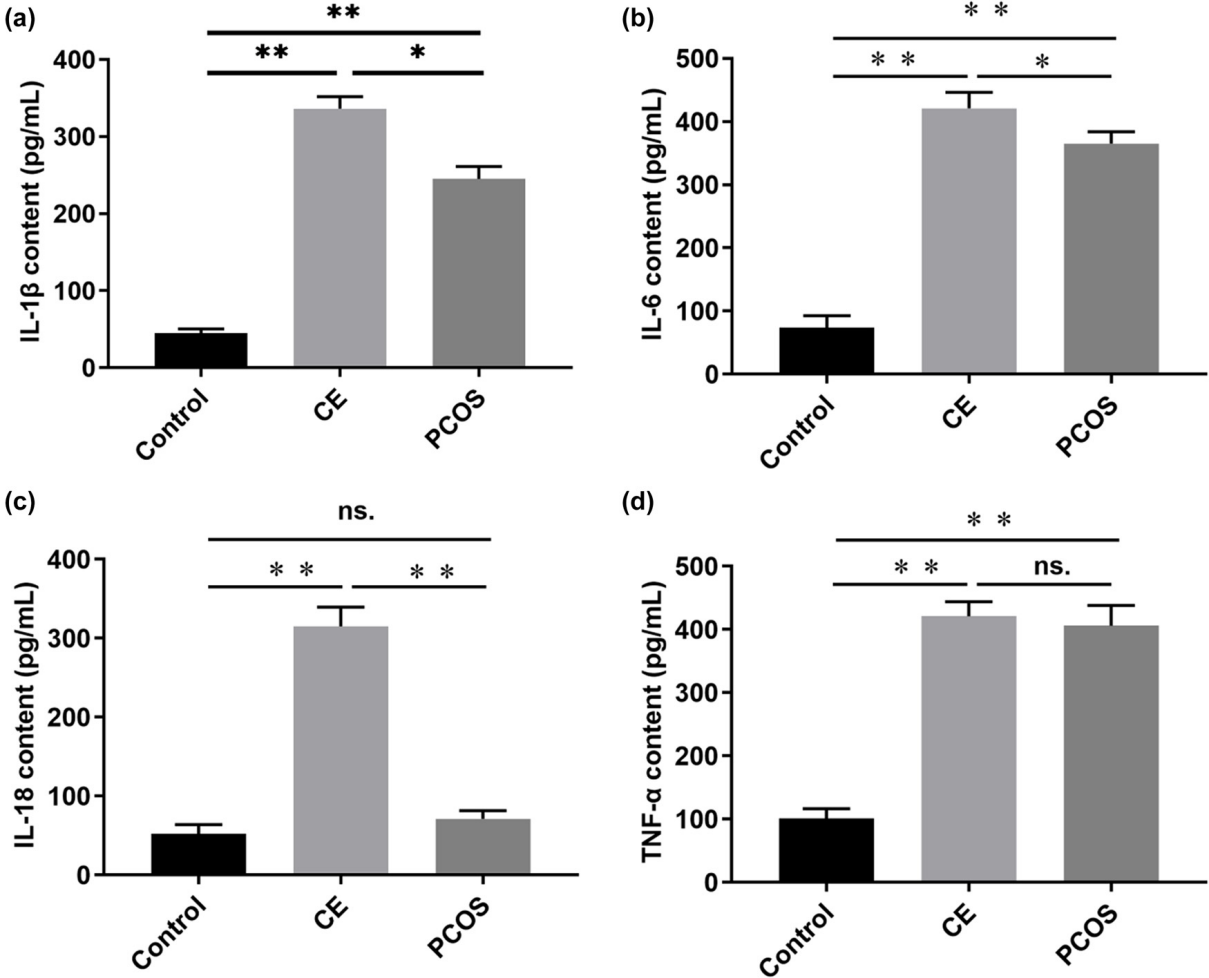
Levels of inflammatory cytokines in endometrial tissue. The control group is black (*n* = 10), the CE group is light grey (*n* = 10), and the PCOS group is dark gray (*n* = 10). *P < 0.05, **P < 0.01. (a) Mean value (bars) ± SD (whiskers) indices secretion levels of IL-1β in endometrial tissue. (b) Mean value (bars) ± SD (whiskers) indices secretion levels of IL-6 in endometrial tissue. (c) Mean value (bars) ± SD (whiskers) indices secretion levels of IL-18 in endometrial tissue. (d) Mean value (bars) ± SD (whiskers) indices secretion levels of TNF-α in endometrial tissue.

ELISA results demonstrated that, compared to the control group, the secretion levels of IL-1β, IL-6, IL-18, and TNF-α in the endometrial tissue of the CE group were significantly elevated (*P* < 0.01). In the PCOS group, the secretion levels of IL-1β, IL-6, and TNF-α were significantly increased relative to the control group (*P* < 0.01), whereas the increase in IL-18 levels was slight and not statistically significant (*P* > 0.05). Furthermore, when comparing the PCOS group to the CE group, the secretion levels of IL-1β, IL-6, and IL-18 were significantly lower (*P* < 0.05), and the reduction in TNF-α levels was minor and not statistically significant (*P* > 0.05). Each group consisted of ten cases (Figure 6).

### 4.6 Protein expression levels of ERS-related molecules in endometrial tissues

**Figure 7:**
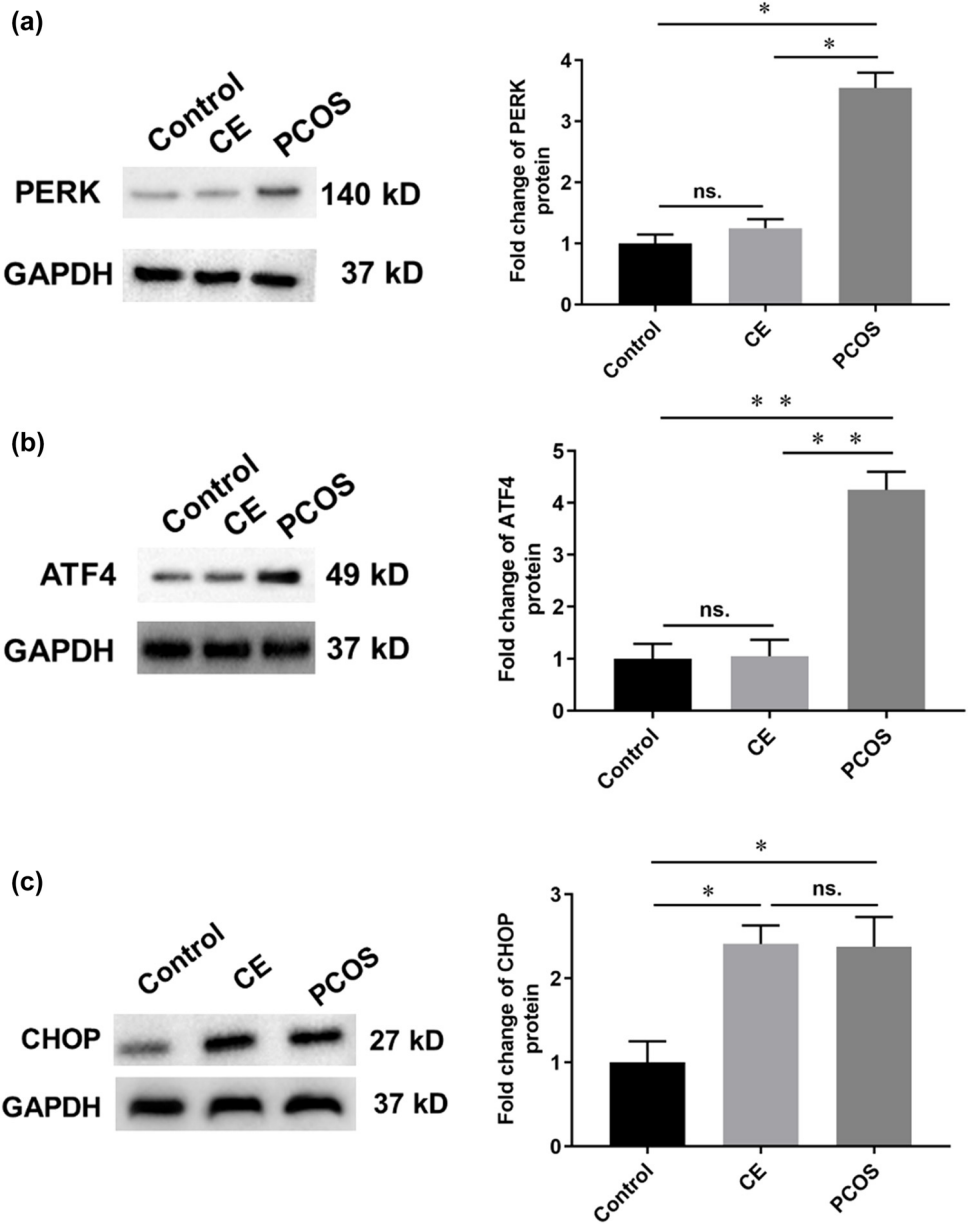
Protein expression levels of ERS-related molecules in endometrial tissues. The control group is black (*n* = 10), the CE group is light grey (*n* = 10) and the PCOS group is dark gray (*n* = 10). *P < 0.05, **P < 0.01. (a) Mean value (bars) ± SD (whiskers) indices protein expression levels of PERK in endometrial tissues. (b) Mean value (bars) ± SD (whiskers) indices protein expression levels of ATF4 in endometrial tissue. (c) Mean value (bars) ± SD (whiskers) indices protein expression levels of CHOP in endometrial tissues.

The Western blot analysis revealed that, in the endometrial tissues of patients with CE, the protein expression levels of PERK and ATF4 did not significantly differ from those in the control group (*P* > 0.05); however, CHOP expression was significantly elevated (*P* < 0.05). In contrast, endometrial tissues from PCOS patients exhibited significant upregulation of the ERS-related proteins PERK, ATF4, and CHOP compared with the control group (*P* < 0.05). Furthermore, when comparing the CE and PCOS groups, the expression levels of PERK and ATF4 were significantly higher in the PCOS patients (*P* < 0.05), whereas the protein expression of CHOP did not show a significant difference (*P* > 0.05). Each group consisted of ten cases (Figure 7).

## 5 Discussion

### 5.1 Results of clinical data analysis

PCOS is a common gynecological endocrine disorder among women of childbearing age, frequently associated with obesity, metabolic syndrome, insulin resistance, and other conditions [18,19]. Numerous studies have shown that individuals with PCOS exhibit elevated levels of subclinical chronic inflammatory markers, a state distinct from the acute inflammatory response typically induced by bacterial or viral infections. This low-grade chronic inflammation, characterized by persistent immune activation, leads to increased production of cytokines such as IL-1, IL-6, IL-8, and TNF-α [20,21]. In the present study, the prevalence of CE was significantly higher in women with PCOS compared to those without PCOS (44.44% vs 22.26%), with endometrial hyperemia being the predominant manifestation. Notably, the positive rate of CD138 in patients with endometrial hyperemia was the lowest (10.14%), a rate that differed significantly from those observed in the micropolyp (62.50%) and edema-hyperplasia (73.87%) groups. Despite comparable embryo quality and normal endometrial thickness on the day of transfer, the clinical pregnancy rate in women with PCOS was lower (33.96% vs 56.58%), and the miscarriage rate was higher than in the non-PCOS cohort. These findings indicate that CE may compromise endometrial receptivity in PCOS patients, particularly among those with obesity, thereby contributing to implantation failure and early pregnancy loss. Based on a review of the literature, we speculate on two potential mechanisms: First, CE may trigger the exudation of inflammatory mediators, leading to endometrial gland dysplasia and disruption of the endometrial microenvironment [22,23]. Second, CE might induce an imbalance in cytokine expression and immune function, thereby impairing the tissue compatibility between the embryo and the endometrium. For example, Schatz’s study demonstrated that during the embryo implantation window, endometrial recruitment of immune cells – such as natural killer cells, macrophages, and T cells – is essential for establishing endometrial receptivity [24]. Similarly, Wang WJ’s study found that patients with CE exhibit elevated levels of Th17 cells and reduced levels of Treg cells, suggesting that CE compromises the local immune microenvironment, undermines endometrial tolerance, and ultimately leads to implantation failure and early pregnancy loss [25].

In the hysteroscopic endometrial hyperemia group, patients exhibited a lower CD138 IHC positivity rate than anticipated, suggesting that endometrial hyperemia may not necessarily indicate an infectious state. Drawing on Cicinelli’s study and our own data analysis [26,27,28], we propose that CE can be classified into two subtypes: one associated with microbial infection and the other with aseptic inflammation. Specifically, the presence of micro-endometrial polyps and endometrial edema-hyperplasia under hysteroscopy appears indicative of microbial infection, while endometrial hyperemia is more consistent with aseptic inflammation. Furthermore, an analysis of patient demographics revealed a higher rate of primary infertility in the hyperemia group compared to the other groups, whereas the secondary infertility rate was elevated in the micro-endometrial polyp and edema-hyperplasia groups. These observations suggest that patients in the latter groups, who likely have a history of pregnancy events (such as spontaneous or induced abortion or childbirth), may be predisposed to an increased risk of uterine infection.

In previous studies, the reported incidence of CE has varied considerably, accompanied by substantial global differences in diagnostic approaches, which adversely affect both diagnosis and treatment. Our research suggests that these discrepancies may be attributed to several factors. First, the heterogeneous patient populations enrolled across studies have led to significant variability. In particular, prior investigations did not differentiate among the three inflammatory manifestations observed under hysteroscopy, instead amalgamating them into a single category. Consequently, distinctions between endometrial hyperemia and cases characterized by micropolyps and edema hyperplasia were not clearly delineated. This inconsistency in categorizing patients with endometrial hyperemia, micropolyps, and edema hyperplasia likely contributes to considerable deviations in CE diagnosis when comparing pathological assessments with CD138 IHC findings.

### 5.2 Results of clinical trials

Lipid distribution was evaluated in endometrial tissue sections using oil red O staining. The results revealed that both the proportion and volume of lipid droplets in the endometrial epithelium and interstitial tissues were significantly increased in the PCOS group, indicating lipid accumulation in the endometrium of these patients. Adipose cell hypertrophy and hyperplasia contribute to the excessive growth of adipose tissue; when neovascularization does not keep pace with this expansion, localized hypoxia ensues. This hypoxia may trigger adipocyte apoptosis, leading to the release of inflammatory chemokines such as monocyte chemokines that attract monocyte-derived macrophages into the tissue. In conjunction with adipocytes, these macrophages enhance the secretion of inflammatory factors including IL-1, IL-6, IL-18, and TNF-α, thereby initiating a chronic inflammatory response [29].

In this study, ELISA was used to quantify inflammatory cytokines in endometrial tissues. The findings demonstrated that the secretion levels of IL-1β, IL-6, and TNF-α were significantly elevated in the PCOS group (*P* < 0.05), suggesting that the endometrium in PCOS patients exists in a state of chronic inflammation. Additionally, RT-qPCR analysis showed that the mRNA expression levels of HIF-1α, VEGF, and EPO were significantly increased in the endometrial tissues of these patients (*P* < 0.05). Under hypoxic conditions, upregulation of HIF-1α activates the transcription of genes encoding VEGF, EPO, and other proteins that enhance oxygen transport and facilitate tissue adaptation to hypoxia. The production of EPO under hypoxia promotes erythropoiesis and increases blood oxygen-carrying capacity. This adaptive response may explain the negative CD138 immunohistochemical staining observed in cases of endometrial hyperemia among PCOS patients.

At this point, we have established that the hyperemic endometrium is indeed experiencing chronic inflammation. However, in conjunction with previous clinical studies, the positive detection rate for CD138 IHC is the lowest in patients with endometrial hyperemia observed during hysteroscopy. Therefore, we hypothesize that the hyperemic state observed in the endometrium of patients with PCOS may not be due to an infectious process but rather represents a form of aseptic inflammation. We will further examine and analyze this hypothesis in detail.

ERS is a key unfolded protein response (UPR) defense mechanism in eukaryotic cells. This protective stress response mitigates the abnormal aggregation of proteins by activating the UPR. However, prolonged or severe ERS can trigger apoptosis. Studies have demonstrated that, in obese mice fed with a high fat diet, significant accumulation of unfolded or misfolded nascent proteins occurs in the ER of oocytes due to lipid accumulation. This accumulation disrupts Ca^2+^ homeostasis, impairs normal ER function, and ultimately induces oocyte apoptosis [30]. The most critical response to ERS is the UPR, which primarily involves the glucose-regulated protein 78 (GRP78/BIP) and three protein sensors. GRP78/BIP, located in the ER and having an approximate molecular mass of 78 kDa, serves as a key regulator of ERS. The three sensors – PERK, activating transcription factor 6 (ATF6), and inositol-requiring enzyme 1 (IRE1) are also situated in the ER and are essential for detecting ERS and propagating downstream signaling, thereby earning the designation of ERS sensors. Under normal conditions, GRP78/BIP binds to these UPR proteins, preventing their activation and the subsequent transmission of UPR signals to the nucleus. However, when ERS occurs, GRP78 dissociates from these sensors, resulting in their activation and the initiation of a cascade of stress response events. The UPR plays a dual role in cellular physiology. Under conditions of transient ERS or mild stimuli, the ER activates the UPR, facilitating proper protein folding and restoring cellular homeostasis, thereby reducing dysfunction. In contrast, prolonged or intense ERS can overwhelm the compensatory capacity of the UPR, leading to apoptosis and a cascade of pathological effects. Sustained ERS diminishes the UPR’s adaptive capability, causing irreversible cellular damage and triggering apoptosis. Additionally, all three branches of the UPR contribute to the activation of the NF-κB signaling pathway following ERS induction, which in turn modulates pro-inflammatory cytokine gene expression and the inflammatory response. Inhibition of ERS-related pathways has been observed to reduce levels of inflammatory factors, such as caspase-1, IL-1, and IL-8, highlighting ERS’ crucial role in mediating inflammatory responses [31]. Researchers have conducted comprehensive studies on the interplay between the UPR and ERS to elucidate the underlying response mechanisms, revealing that the onset and progression of numerous chronic diseases are associated with UPR [32]. Adipose tissue dysfunction has been implicated in the metabolic and inflammatory abnormalities observed in PCOS [16], while ERS activation in granulosa cells contributes to the pathophysiology of PCOS through multiple mechanisms [33]. In the present study, RT-qPCR was employed to assess the mRNA expression levels of ERS-related molecules in endometrial tissues. The RT-qPCR results demonstrated that, relative to the control group, the mRNA expression levels of ATF4, GRP78, and HSP70 in the endometria of PCOS patients were significantly elevated (*P* < 0.05). These findings suggest that hypoxia-induced ERS in the endometrium of PCOS patients may impair endometrial receptivity and contribute to a reduced pregnancy rate.

## 6 Conclusion

In summary, this study addresses two primary questions. First, our findings indicate that the prevalence of CE is higher in women with PCOS compared to those without PCOS. Notably, the predominant manifestation of CE in PCOS women is endometrial hyperemia, with the lowest positive rate of CD138 IHC observed in patients exhibiting this condition. Second, we hypothesize that endometrial hyperemia in women with PCOS may be caused by endometrial hypoxia, which subsequently induces ERS in the endometrium and promotes the secretion of inflammatory factors. Although these changes are not attributable to microbial infections, the precise etiology remains unclear and warrants further in-depth research. Future studies will involve stratifying PCOS patients based on body weight and testosterone levels, given that elevated testosterone may also contribute to CE. Ideally, comparative analyses of endometrial samples obtained before and after weight loss in PCOS patients would help determine whether there is a reduction in ERS.
